# Feedback Methods for Vector Measurements Using an All-Optical Atomic Magnetometer

**DOI:** 10.3390/s23094263

**Published:** 2023-04-25

**Authors:** Michael Bulatowicz, Jonas Tost, Thad G. Walker

**Affiliations:** Department of Physics, University of Wisconsin-Madison, Madison, WI 53706, USA

**Keywords:** magnetometers, vector measurement, feedback

## Abstract

In this work, we look to compare three methods of feedback for the ultimate purpose of measuring the transverse vector components of a magnetic field using a synchronous light-pulse atomic scalar magnetometer with a few tens of fT/$\sqrt{\mathrm{Hz}}$ sensitivity in Earth-field-scale magnetic environments. By applying modulation in the magnetic field to orthogonal axes, the respective vector components may, in principle, be separated from the scalar measurement. Success of this technique depends in significant part on the ability to measure and respond to these perturbations with low measurement uncertainty. Using high-speed least-squares fitting, the phase response of the atomic spins relative to the first harmonic of the optical pump pulse repetition rate is monitored and correspondingly adjusted into resonance with the natural Larmor precession frequency. This paper seeks to motivate and compare three distinct methods of feedback for this purpose. As a first step toward the full development of this technique, the present work uses a simplified version with modulation applied only along the bias field. All three methods investigated herein are shown to provide results that match well with the scalar magnetometer measurements and to depend on both the applied modulation amplitude and optimal feedback response to achieve low relative uncertainty.

## 1. Introduction

Magnetometry has been approached and realized in many different ways, and is foundational to many modern technologies. Ultrahigh-sensitivity magnetometers have applications in a number of fields, including biomagnetism [1,2,3,4,5,6,7], gravitational wave detection [8], geosensing [9], dark matter searches [10], astrophysics [9,11], infrastructure monitoring [12], materials inspection [13], navigation aiding [14], and so on. Improvements in production techniques and quality of components, accompanied by increased portability and sensitivity, has resulted in increased interest in optical atomic magnetometers, often called “optically pumped magnetometers” (OPMs). Recent work has demonstrated their ability to achieve sub-fT/$\sqrt{\mathrm{Hz}}$ sensitivities in near-zero field environments [15] and 3-axis vector sensitivity in near-zero-field environments [4,5,16,17,18], μT-level environments with relatively small vector components orthogonal to the bias field [19], and yet more advances have been made in extending the operational range of spin-polarized optically pumped magnetometers into the Earth-field regime [20,21,22,23].

The current gold standard in ultrahigh-sensitivity vector magnetometry in the Earth field is the superconducting quantum interface device (SQUID) magnetometer. The SQUID magnetometer is inherently a vector sensor and detects field projections along its sensitive axis. Furthermore, its capacity to achieve ultrahigh sensitivity has been demonstrated in Earth-field environments [24]. In contrast, optically pumped atomic magnetometers capable of operating in Earth-field-scale magnetic fields typically function by measuring the Larmor precession frequency of atomic spins of vapor-phase alkali metals [25] or helium [26] in the presence of magnetic fields, and thus are inherently scalar field sensors. However, methods have been proposed for the measurement of the vector components of the incident field using these sensors, with at least one example making use of microwave polarization reconstruction [27]. Thus, 3-axis magnetic sensing using an OPM does not inherently require three physically separate devices which would add complexity and potentially degrade the measurement accuracy for nearby sources of the magnetic field. Additionally, unlike SQUID magnetometers, OPMs do not require cryogenic cooling, thus reducing operating costs and improving portability.

While in many settings, scalar field measurements are sufficient, full knowledge of vector components provides additional insight into the ambient field, which is useful for many applications, such as magnetoencephalography [4,5], magnetometer-based tracking of a magnetic object (for example [28]), and the enhancement of magnetic-field-based navigation aiding, using a greater portion of the information available in Earth’s magnetic field as compared to scalar measurement alone [29]. There is further interest in utilizing vector component information to correct for inherent “heading errors” in alkali-based magnetometers, which results from the non-zero nuclear spin of alkali atoms [30]. Magnetic vector component measurement further enables the measurement of the magnetic gradient tensor [31], which may improve the precision and accuracy of, for example, navigation aiding [32].

In this work, a Bell–Bloom [33,34] magnetometer with a single optical axis utilizes the intensity modulation of an optical pump beam along $\hat{x}$ passing through a vapor cell (a 1 cm diameter by 1 cm length internal dimension cylinder containing isotopically enriched ${}^{87}Rb$ and nitrogen buffer gas) to drive the coherent spin precession of ${}^{87}Rb$ spins in an Earth-field-scale magnetic field. The intensity of the optical pump is modulated as a series of short-duty-cycle pulses in a manner similar to that described in [20]. The optical pulse repetition rate is approximately resonant with the natural Larmor precession frequency $\omega_{L}$ of the spins, with a first harmonic component cos(ωt), where $\omega \approx \omega_{L}$. A linearly polarized optical probe beam co-propagating with the pump measures the $\hat{x}$ projection of the spin polarization $\vec{P}$; the linear polarization of the optical probe rotates proportional to $\vec{P}\cdot \hat{x}$.

The polarization rotation of the optical probe beam is measured using a balanced polarimeter with a custom differential transimpedance amplifier circuit, and the resulting electrical signal is digitized using an analog-to-digital converter with 14-bit resolution at 250 million samples per second. An FPGA reads the digitized signal and performs real-time least-squares fitting of the observed polarimeter signal to measure the phase response of atomic spins relative to a digital reference model consisting of cos(ωt) and sin(ωt) components synchronized to the optical pump pulses. Closed-loop feedback adjusts the pump pulse repetition rate to drive $\omega_{p}\to \omega_{L}$ by any of the three feedback methods as described herein.

In general, by applying three spatially orthogonal oscillating magnetic fields to the inherently scalar magnetometer, each vector component of the incident magnetic field may be extracted from the resulting modulation of the overall measured measured magnetic field [35], herein based on the natural Larmor precession frequency of the spins. For each vector direction $\hat{i}$, the magnetic field component along $\hat{i}$ takes the form $B_{i}\left(t\right)=B_{i,0}\left(t\right)+B_{i}^{\prime }sin\left(\omega_{i}t\right)$. Given three orthogonal vector directions ($\hat{x}$, $\hat{y}$, and $\hat{z}$ in the instrument reference frame, for example) the total magnetic field $\vec{B}$ observed by the instrument is

(1) $$\begin{array}{c} |\vec{B}|=\sqrt{\sum_{i}B_{i}^{2}} \end{array}$$

The squared magnitude along each $\hat{i}$ can be written as the square of the sum of the low-frequency component $B_{i,0}$ and modulation component $B_{i}^{\prime }sin\left(\omega_{i}t\right)$:

(2) $$\begin{array}{cc} B_{i}{\left(t\right)}^{2}={\left(B_{i,0}+B_{i}^{\prime }sin\left(\omega_{i}t\right)\right)}^{2} & \\ =B_{i,0}{\left(t\right)}^{2}+2B_{i,0}\left(t\right)B_{i}^{\prime }sin\left(\omega_{i}t\right)+\frac{{B_{i}^{\prime }}^{2}}{2}\left(1-cos\left(2\omega_{i}t\right)\right) \end{array}$$

As a result, it is possible to find both $B_{i,0}$ and $B_{i}^{\prime }$ by demodulating the square of the measured magnetic field (Larmor frequency as observed by way of the pump pulse repetition rate) at the first and second harmonics of the applied fields $B_{i}^{\prime }sin\left(\omega_{i}t\right)$. As the measured quantity is specifically the pump pulse repetition rate, the success of this method depends on the ability to measure and respond to each applied ($B_{i}^{\prime }sin\left(\omega_{i}t\right)$). A top-level block diagram of the implemented algorithm is shown in Section 3. This paper seeks to compare three feedback methods for tracking the Larmor frequency, with the ultimate goal of minimizing errors in vector calculations for a full three-axis implementation. As a first step toward this goal, the present work implements only a single modulation field, superimposed on the bias field such that $|\vec{B}{|}^{2}={\left(B_{z,0}+B_{z}^{\prime }sin\left(\omega_{z}t\right)\right)}^{2}$, significantly simplifying the analysis and interpretation of results as compared to a full 3-axis implementation.

Note that in Equation (2), the assumption is made that the applied magnetic fields form an orthogonal basis set. In reality, effects such as build tolerances, mechanical stresses, differential thermal expansion, and so on guarantee that there will exist some finite deviation from orthogonality of the magnetic field coils producing these fields. As shown in [36], one may form an orthogonal basis set from the three magnetic field coils using a carefully measured mapping matrix.

## 2. Experimental Apparatus and Methods

**Figure 1:**
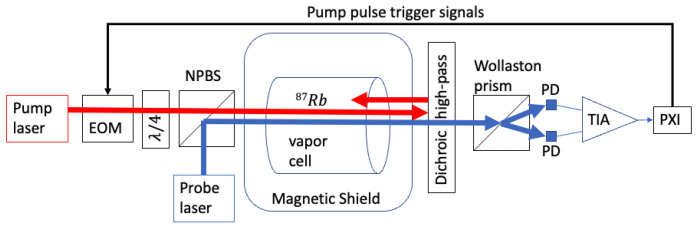
Block diagram of the experimental apparatus. The 795 nm optical pump beam pulses are generated using a continuous-wave laser with a custom shuttering system based on an electro-optic modulator (EOM). The pump beam is circularly polarized using a quarter-wave plate (λ/4). A non-polarizing beam splitter cube (NPBS) combines the optical pump and probe beams such that they co-propagate into the magnetically shielded enclosure and through the ${}^{87}Rb$ vapor cell. On the opposite side of the magnetically shielded enclosure is a dichroic high-pass filter designed to reflect the optical pump beam back toward the vapor cell and transmit the optical probe beam to a Wollaston prism, which functions as a polarization beam splitter; S and P polarization components of the probe beam are each sent to a respective photodetector (PD). The Wollaston prism is oriented at approximately 45 degrees relative to the unrotated plane of polarization of the probe beam such that the photodetectors generate approximately equal photocurrents in the absence of probe beam polarization rotation. The observed photocurrents are differenced and converted to a differential voltage signal using a custom transimpedance amplifier (TIA). Finally, the signal is read into the NI PXI-based digital system; feedback controls the optical pump pulse repetition rate by way of signals triggering the EOM.

A block diagram of the apparatus is shown in Figure 1. At the core of the experiment is a cylindrical glass vapor cell (1 cm internal diameter by 1 cm internal length; chosen for size compatibility with the electrical resistive heaters already on hand in our lab from previous work [3]) containing a droplet of ${}^{87}Rb$ and 0.8 amagat $N_{2}$. The vapor cell is surrounded by ceramic RF heating coils that are designed to minimize induced magnetic fields [37] and thermal insulators consisting of aerogel sheets to maintain a ${}^{87}Rb$ vapor pressure of approximately 3 × $10^{12}$ cm${}^{-3}$. In accordance with [37], each RF heating coil is a planar 3-layer thick-film-on-substrate ceramic circuit board with a magnetic 16-pole winding pattern around the substrate perimeter, designed for minimum self-inductance (minimum induced magnetic field and minimum reactive impedance); the vapor cell is surrounded by a pair of these heating coils, oriented opposite to each other for a net 32-pole RF magnetic coil pattern surrounding the vapor cell.

Perturbation of the spins induced by the residual magnetic fields that are generated by the current flowing through the coils is further reduced by way of driving the current at approximately 2 MHz with a dissipated heat power of approximately 0.3 W, far off the resonance relative to the spin precession frequency and far outside the response bandwidth of the magnetometer. In principle, one may also heat the vapor cell without any local sources of magnetic field using a laser of an appropriate wavelength to be absorbed by the glass of the vapor cell itself or an appropriate attached optical absorption element [38]. The insulated vapor cell assembly is housed inside a 3D printed custom mount within a 4-layer magnetic shield (Twinleaf MS-2), which provides access to the co-propagating probe and pump lasers to pass through the shield and vapor cell assembly without any optical elements internal to the magnetic shield. Within the shielding are integrated magnetic field coils capable of generating both uniform and gradient fields.

A Twinleaf CSUA-1000 current supply drives the current through one of the uniform field coils to generate an ultra-low noise bias field on the order of 29 μT, allowing measurement and verification of the instrument noise floor down to approximately 10 fT/$\sqrt{\mathrm{Hz}}$, an impressive fractional noise value of roughly −190 dB/$\sqrt{\mathrm{Hz}}$. Using a function generator to provide a sinusoidal driving signal, perturbations up to 267 nT can be superimposed on the bias field through the CSUA modulation input; this was insufficient for the largest-amplitude modulation signals applied in this experiment, so the CSUA-1000 is placed in parallel with a custom current supply circuit capable of significantly larger modulation fields but with a white noise floor of approximately 60 fT/$\sqrt{\mathrm{Hz}}$ and a 1/f noise limit of approximately 2 pT/$\sqrt{\mathrm{Hz}}$ at 0.1 Hz, thereby dominating the observed magnetic noise spectrum.

The atomic vapor is polarized using a circularly polarized pump laser tuned near to the 795 nm D1 line of ${}^{87}Rb$ and pulsed with a short duty cycle at a repetition rate approximately equal to the natural Larmor precession frequency in the magnetic bias field. The pump pulse repetition rate is controlled using a direct digital synthesis (DDS) scheme with up to 64 bits of precision; the DDS is internal to a FPGA (field-programmable gate array), which outputs logic control signals to drive the state of the optical pump beam (on or off) via an electro-optical modulator (EOM). The closed-loop feedback described below updates the DDS frequency (and phase, if applicable) to drive the pump pulse repetition rate to the natural Larmor precession frequency in the presence of perturbations to the scalar field observed by the sensor, and the DDS frequency is captured and recorded as representative of the Larmor precession frequency.

Each pump pulse exhibits an “on” state intensity of roughly 10 mW, incident on the vapor cell, and the magnetometer sensitivity under our normal operating conditions is observed to maximize at a duty cycle of 0.07, corresponding to 0.7 mW time-average optical pump power incident on the vapor cell, as measured by a Coherent^®^ LaserCheck™ optical power meter. Between pulses, the polarized spins precess about the external bias field at the Larmor frequency $\omega_{L}=\gamma B$ such that the spin polarization relative to the bias field axis can be written as $P\left(t\right)=P_{⊥}cos\left(\omega_{L}t+\phi \right)+P_{\|}$. For this experiment, the bias field is nominally orthogonal to the optical pump beam such that $P_{\|}\to 0$. The pump pulse repetition rate is tuned near the first harmonic of the natural Larmor precession frequency of the spins in the scalar magnetic field observed by the instrument.

A linearly polarized CW (continuous wave) probe laser passes through the cell to track the spin-dependent index of refraction of the ${}^{87}Rb$ vapor via Faraday rotation. This polarized light is detuned multiple linewidths away from the broadened ${}^{87}Rb$$D_{2}$ optical resonance near 780 nm in order to reduce the spin relaxation effects of photon scattering from the probe light and exhibits an optical power of 3 mW incident on the polarimeter. The angle θ of rotation of the linear polarization of the detected probe light then follows $\theta \left(t\right)\propto N\hat{x}\cdot P\left(t\right)$, where *N* is the number of spins interacting with the probe beam and the probe is propagating along $\hat{x}$. The resulting polarization rotation angle is measured with a balanced polarimeter; for small polarization rotation angles, the differential photocurrent is proportional to the rotation angle. Given a pair of photodetectors in the balanced polarimeter with photocurrents $I_{1}$ and $I_{2}$,

(3) $$\begin{array}{c} \theta \approx \frac{1}{2}\frac{I_{1}-I_{2}}{I_{1}+I_{2}} \end{array}$$

The noise output from the polarimeter is consistent with the photon shot noise limit. The polarization angle noise δθ in a 1 Hz bandwidth for small angles is based on the electrical current shot noise from the photodetectors, given elementary charge *q* on a single electron:

(4) $$\begin{array}{c} \delta \theta \approx \frac{1}{2}\frac{\sqrt{2q(I_{1}+I_{2})}}{I_{1}+I_{2}}=\frac{1}{2}\sqrt{\frac{2q}{I_{1}+I_{2}}} \end{array}$$

For a 3 mW probe at 780 nm wavelength, the silicon phototdetectors provide a responsivity of slightly over 0.5 A/W for a total polarization rotation noise of approximately 7 nano-radians per square root Hz (nrad/$\sqrt{\mathrm{Hz}}$). The measured slope of the response ($\frac{d}{dB}\left(I_{1}-I_{2}\right)$) is typically in the range of 3000 to 5000 amps per Tesla; based on Equation (4), at low frequencies ω≪Γ, where Γ is the transverse spin polarization relaxation rate, the photon shot noise limit of magnetic field detection is therefore at or below 7 fT/$\sqrt{\mathrm{Hz}}$, well below the observed total magnetic noise.

The differential photocurrent signal is converted to voltage with a custom differential transimpedance amplifier; the voltage signal is sent to an analog-to-digital converter (ADC) input of a NI PXIe-5171 FPGA reconfigurable PXI oscilloscope module (14 bits per sample, 250 million samples per second, 125 MHz FPGA clock rate). The on-board Kintex-7 410T FPGA on the NI PXIe-5171 module is used for the real-time signal processing, feedback, and generation of optical pump pulse trigger signals (Figure 1), using custom LabVIEW-based FPGA firmware. Real-time least-squares demodulation of the observed voltage signal based on the driven pump pulse repetition rate allows for the recovery of ϕ with 47 bit precision in 0.43 μs, making it a useful tool for tracking and correcting repetition rate errors $\delta \omega =\omega_{L}-\omega$.

The method of pulsing the optical pump beam at a rate of approximately $\omega_{p}=\omega_{L}$ effectively pumps the spins in their rotating reference frame; the Fourier transform of a sequence of square pulses contains the pulse repetition rate as a major component. Starting from the Bloch equation for spins in a magnetic field, it can be shown [22] that the observed phase difference ϕ between the spins and the pump pulses near resonance corresponds to the difference δω between the pump pulse repetition rate and the natural Larmor precession frequency of the spins, and further includes contributions from the phase response $\phi_{pol}$ of the polarimeter circuitry and the electronics system latency δt:

(5) $$\begin{array}{c} \phi =tan^{-1}\left(\Gamma \delta \omega \right)+\phi_{pol}+\omega \delta t \end{array}$$

In the limit where Γδω≪1, the phase shift resulting from δω is directly proportional to δω, and the response is assumed to be linear such that

(6) $$\begin{array}{c} \phi \to \Gamma \delta \omega +\phi_{pol}+\omega \delta t \end{array}$$

Each of the feedback methods investigated herein is designed to correct the pump pulse repetition rate directly using the measured phase ϕ as the error signal driving the loop. The phase shift (accumulated phase error) δϕ is measured over some period of time, in this case typically a single precession cycle of the spins. Noting that $\frac{d}{dt}\delta \phi =\delta \omega$, where δω is the difference between the angular frequency of precession of the spins and the pump pulse repetition rate, it becomes clear that feeding back to the frequency based on the measurement of phase inevitably leads to a precession–frequency-dependent feedback gain component that can lead to instability in the closed-loop response upon increasing the scalar field magnitude and adversely affect bandwidth upon decreasing the scalar field magnitude. This gain component can be mitigated by any of a number of different methods, such as feeding back based on the product δϕT, where δϕ is again the accumulated phase error and *T* is the measurement period, rather than feeding back based directly on δϕ. The signal-to-noise ratio (SNR) for the measurement of oscillating magnetic fields at a frequency $\omega_{osc}$ is based on the SNR at very low frequencies ($SNR_{0}$; $\omega_{osc}\ll \Gamma$), the frequency of interest $\omega_{osc}$, and the SNR bandwidth $\omega_{0}=\Gamma /\pi$; in the limit of high gain at $\omega_{osc}$, this SNR is independent of the feedback method chosen. Thus, the instrument exhibits a “signal-to-noise ratio bandwidth” that is essentially independent of the closed-loop −3 dB response bandwidth. At low frequencies, then, each feedback method is expected to exhibit effectively identical SNR.

(7) $$\begin{array}{c} SNR\left(\omega_{osc}\right)\approx \frac{SNR_{0}}{\sqrt{1+\frac{\omega_{osc}^{2}}{\omega_{0}^{2}}}} \end{array}$$

### 2.1. PI Feedback

**Figure 2:**
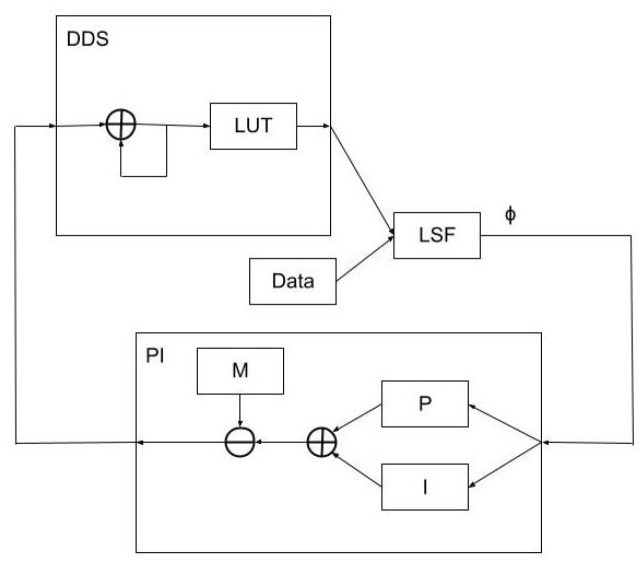
A top-level block diagram of the PI feedback scheme. The block labeled DDS is the digital representation of the Rb spin precession phase and frequency and includes a look-up table (LUT) to convert the DDS phase word into a sinusoidal model of the spin precession; its output is compared to the incoming digitized precession signal (data) in a least-squares filter (LSF) that outputs the value of δϕ. δϕ is then used to drive the PI gain stage (block labeled PI). The result of the PI calculation modifies the DDS phase increment word M.

The input to the PI (proportional plus integral) gain stage in the first of the three closed-loop feedback schemes discussed herein takes the δϕ value calculated by the least-squares algorithm and continuously calculates updates to the pump pulse repetition rate as the sum of the proportional and integral gain components. The proportional gain component is a simple multiple of δϕ, and the integral gain component multiplies δϕ by a second gain and sends the result to a digital accumulator. A top-level block diagram is shown in Figure 2. For this work, the PI gain stage is tuned by maximizing the absolute gains while avoiding instability.

### 2.2. Nonlinear Feedback

In general, the accumulation of phase between two sinusoids, such as the precession signal and the reference signal, occurs as in Equation (8):

(8) $$\begin{array}{c} \frac{d\delta \phi (t)}{dt}=\omega_{ref}\left(t\right)-\omega \left(t\right) \end{array}$$

**Figure 3:**
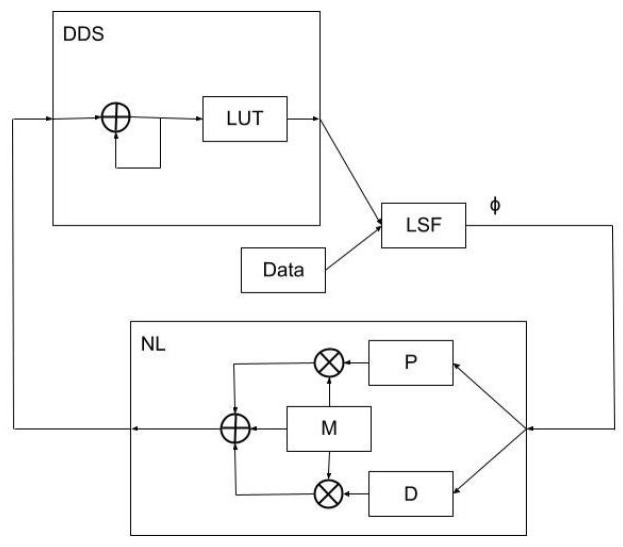
A top-level block diagram of the non-linear feedback scheme.

Meanwhile, Equation (5) indicates that the frequency offset is directly proportional to the tangent of phase rather than proportional to the phase per se; an important point is that Equation (6) holds only for the following cases: (1) in a steady-state condition and (2) only for small phase offsets. One may more accurately capture a portion of the nonlinear phase response to rapid deviations in precession frequency through a slightly more sophisticated method. More generally, a shift in the ambient field is detected as a temporary shift in the precession frequency relative to the reference frequency (Equation (8)). Comparing in discrete time the most recent measurement of phase at time interval *n* (ϕ[n]) to the previous measurement of phase (ϕ[n−1]), based on the time between measurements $T_{ref}\left[n\right]$, one may deduce the shift in resonant frequency from the previous to present measurements:

(9) $$\begin{array}{c} \frac{\phi [n]-\phi [n-1]}{2\pi T_{ref}\left[n\right]}=f_{ref}\left[n\right]-f\left[n\right], \end{array}$$

where $f_{ref}$ is the reference frequency to which the actual data are being compared (the output of the DDS block in Figure 3). Making a first-order approximation of the time derivative of Equation (9) and inserting the result into Equation (8), it is possible to solve for the present actual resonant frequency f[n] of the spins (Equation (9)). One may then predict a first-order approximation of the expected precession frequency f[n+1] at the next measurement interval and preemptively update the model ($f_{ref}\left[n+1\right]$) by way of the DDS phase increment word *M*. From this, a feedback scheme is constructed, which has a similar form to the PI gain stage (Equation (10)):

(10) $$\begin{array}{c} f_{ref}\left[n+1\right]=f_{ref}\left[n\right]\left(1-K_{p}\left(\phi \left[n\right]-\phi \left[n-1\right]\right)-K_{i}\phi \left[n\right]\right) \end{array}$$

A proportional term ($K_{p}$) approximates the derivative between consecutive phase terms, while an integral term ($K_{i}$) approximates the derivative to zero phase. Conceptually, this scheme can be seen as a translation from a derivative-proportional (DP) controller into a PI (proportional-integral) controller via a frequency-dependent multiplicative factor. So, this method captures both the phase deviation itself and a multiple of its time derivative, and therefore generates a gain response that is nonlinear in phase deviation. A top-level block diagram is shown in Figure 3. For this work, the gain stage is tuned to maximize the closed-loop −3dB response bandwidth at approximately 17.5 kHz.

### 2.3. Hybrid Self-Oscillator

Based on Equation (5), it can be understood that the control of the pump pulse repetition rate is required in order to drive δϕ→0. In a “pure” self-oscillator, the periodic incoming data directly generate the driving signal. Stated another way, rather than driving the frequency in order to alter the phase, the phase of the pump pulses is driven in order to alter the frequency.

In this method, the phase deviation δϕ of the periodic input ∝P(t) directly drives the phase of both the pump pulses and the reference sinusoid. However, the frequency of the reference must be deduced based on the phase to ensure that the drive frequency matches the reference frequency. In discrete terms, the number of steps taken by the DDS accumulator in one period follows as $N=2^{n}/m$, where *m* is the DDS phase word and *n* is the bit width of the accumulator. A non-zero phase can be corrected with a shift in the size of the accumulator with a new phase word $m^{\prime }=\left(2^{n}+K_{i}^{\prime }\phi \right)/N$. Reorganizing these terms results in an updated phase word $m^{\prime }$ and a third feedback scheme:

(11) $$\begin{array}{c} m^{\prime }=m\left(1-K_{i}\phi \right) \end{array}$$

**Figure 4:**
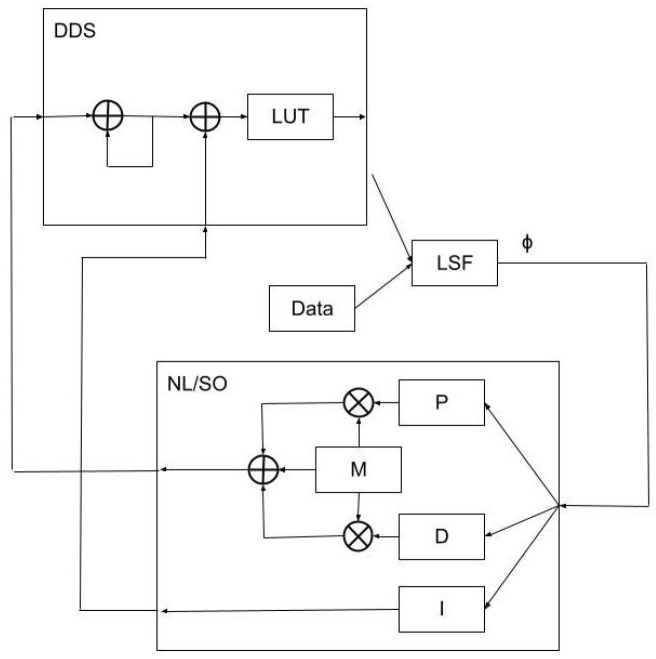
A top-level block diagram of the hybrid self-oscillator feedback scheme.

For a “pure” self-oscillator approach, direct feedback of the measured phase response as a phase shift in the timing of the optical pump pulses now takes the place of the proportional feedback in the PI and nonlinear feedback schemes. For the PI and nonlinear approaches, the proportional feedback term directly modifies the repetition rate of the optical pump pulses rather than directly modifying their phase. However, since the pure self-oscillator feedback approach does not directly influence the closed-loop −3 dB response bandwidth, it was found to be beneficial to implement a combination of both phase and proportional feedback; hence, it was designated as a hybrid self-oscillator based on this mixing of the nonlinear and self-oscillator methods. A top-level block diagram is shown in Figure 4. For this work, the response bandwidth was maximized at approximately 19 kHz.

### 2.4. Feedback Loop Summary

A comparison between the three feedback loop schemes may be understood qualitatively as follows. First, the PI scheme measures the phase difference between the DDS model of the expected precession signal and the actual signal itself and directly uses this phase to update the pump pulse repetition rate (and DDS). The PI scheme is therefore only reactive—it responds to a measured phase deviation and makes corrections. Second, the nonlinear scheme takes advantage of the time derivative of the phase to predict the next observed spin precession frequency and updates the DDS phase increment word accordingly. The nonlinear scheme is therefore both reactive and predictive; it deliberately seeks to predict the next observed signal increment rather than only responding to the presently observed signal increment. Finally, the hybrid scheme takes the nonlinear scheme and adds a direct phase modification of the next pump pulse based on the observed phase of the present signal with respect to the model (DDS). The hybrid scheme is therefore reactive and predictive, and includes an additional correction factor for further improvement of the timing of the optical pump pulses to coincide with the resonant precession of the spins.

While the PI feedback scheme may accurately correct for frequency offsets, for increasing dB/dt, the latency δt in calculating δϕ and updating frequency increasingly limits the phase margin. Predictive modification of the pump pulse repetition rate as in the nonlinear scheme recaptures some of this margin. Meanwhile, a direct adjustment to the phase as in the hybrid self-oscillator feedback mechanization can be expected to allow for faster response times to larger dB/dt, as it does not solely rely on the accumulation of phase inherent in the δϕ error signal calculation that drives the PI and nonlinear schemes. Thus, at larger values of the frequency–magnitude product $\omega_{i}B_{i}^{\prime }$ (i.e., a larger dB/dt) the hybrid scheme can be expected to more closely track the spin precession frequency perturbation induced by the applied oscillating magnetic fields as compared to the PI or nonlinear schemes.

### 2.5. Measurement Uncertainty

As shown in Equation (2), measurement of the vector components of the incident magnetic field requires observation of the first- and second-harmonic components of the square of the measured total magnetic field in the presence of a modulation applied along the axis for which the vector component is to be observed. Uncertainty in the measurement of these components, such as that arising from the finite gain of the feedback loop, will result in uncertainty in the calculated vector component solution. A key point in the evaluation of the effects of uncertainty in the closed-loop response is that the instrument is inherently a scalar magnetometer. Extension of the instrument’s operation to vector magnetic field measurement is achieved through applied modulating magnetic fields and by way of processing the signals in magnetic-field-squared space.

The scalar magnetic field $B_{s}$ is perceived by the closed-loop measurement system as measurement value M. Note that *M* is simply an appropriately scaled version of the DDS phase increment word *m* mentioned above, converted into magnetic field units: the instrument in the present work is a magnetic-field-to-frequency transducer by way of the relationship between the scalar field, frequency, and the gyromagnetic ratio γ of the spins: $\omega =\gamma B_{s}$. The value of *M* includes the actual scalar magnetic field $B_{s}$ at any particular epoch and an additional uncertainty δB, which includes effects from the finite gain of the feedback loop as well as noise and effects from any applied filtering. Thus, the vector measurement portion of the system perceives

(12) $$\begin{array}{c} M^{2}={\left(B_{s}+\delta B\right)}^{2} \end{array}$$

One may expand the $B_{s}$ term as a function of time, defining the static (low frequency) portion of each vector component as $B_{i,0}$ with applied modulation of amplitude $B_{i}^{\prime }$ and frequency $\omega_{i}$:

(13) $$\begin{array}{c} B_{s}^{2}\left(t\right)=\sum_{i}{\left(B_{i,0}+B_{i}^{\prime }cos\left(\omega_{i}t\right)\right)}^{2} \end{array}$$

where $B_{0}$ is the low-frequency component of the incident magnetic field; here, low frequency is defined as lower than $\omega_{i}$. In the general case $i=\{\hat{x},\hat{y},\hat{z}\}$ and it is assumed that modulation is applied along three orthogonal components of the incident magnetic field (i.e., modulations along $\hat{x}$, $\hat{y}$, and $\hat{z}$ in the instrument reference frame). In this work, as a first step toward 3-axis measurement, a single oscillating field is applied along the bias field to simplify analysis and interpretation of the experimental results, reducing Equation (13) to simply

(14) $$\begin{array}{c} B_{s}^{2}\left(t\right)={\left(B_{0}+B^{\prime }cos\left(\omega t\right)\right)}^{2} \end{array}$$

One may separate δB into its Fourier components at integer multiples of ω to provide additional insight:

(15) $$\begin{array}{c} \delta B=\sum_{k=0}^{\infty }\delta B_{k}cos\left(k\omega t+\phi_{k}\right) \end{array}$$

Combining Equations (12), (14) and (15) yields

(16) $$\begin{array}{c} M^{2}=B_{0}^{2}+2B_{0}B^{\prime }cos\left(\omega t\right)+{B^{\prime }}^{2}cos^{2}\left(\omega t\right)\\ +2\sum_{k=0}^{\infty }\left[\delta B_{k}cos\left(k\omega t+\phi_{k}\right)\left(B_{0}+B^{\prime }cos\left(\omega t\right)\right)\right]\\ +{\left(\sum_{k=0}^{\infty }\delta B_{k}cos\left(k\omega t+\phi_{k}\right)\right)}^{2} \end{array}$$

Equation (16) demonstrates that one may measure the amplitude of the applied oscillating field based on demodulation of the square of the scalar field at 2ω (note: $cos^{2}\left(\omega t\right)=\frac{1}{2}\left[1+cos\left(2\omega t\right)\right]$); this result combined with demodulation of the square of scalar field at ω provides a solution for the vector component of magnetic field along the applied oscillating field direction. Equation (16) also clearly demonstrates that the process of squaring the magnetic field will generate the mixing of harmonics; particularly relevant are mixed components that result in observed frequency content at ω and 2ω. Significant benefits can therefore be realized through appropriate filtering of the scalar field before and after the squaring operation, prior to demodulation.

**Figure 5:**
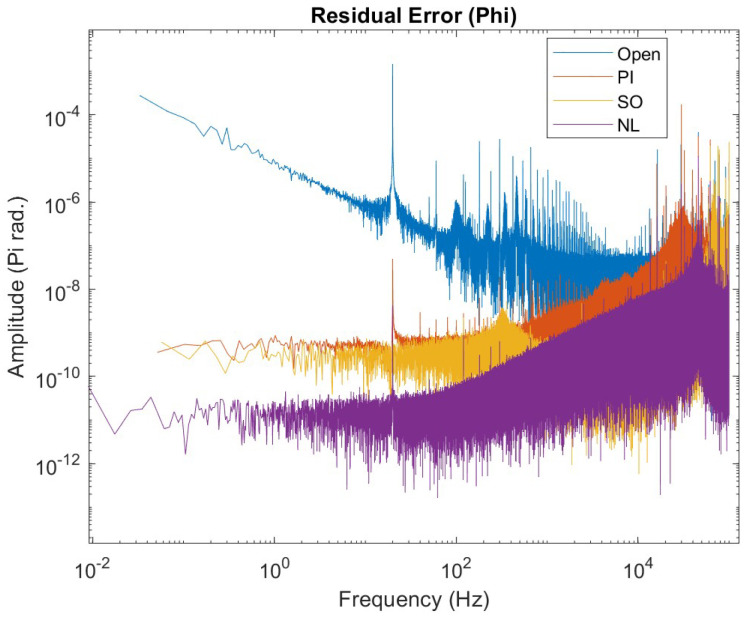
A comparison of residual errors between open loop and closed loop operation in a 29 μT bias magnetic field (200 kHz precession frequency) with a 20 Hz, 0.534 nT (3.74 Hz precession frequency perturbation amplitude) oscillation.

This work utilizes a sinc (in frequency) filter prior to squaring; a bandpass filter at ω prior to squaring for detection of the 2ω component; and bandpass filters at ω and 2ω after squaring (Figure 5). In this work, $B^{\prime }\leq$ 534 nT (3740 Hz precession frequency perturbation amplitude), while $B_{0}\approx$ 29,000 nT (200 kHz precession frequency amplitude); thus, in Equation (16), it becomes apparent that ${B^{\prime }}^{2}\ll 2B_{0}B^{\prime }\ll B_{0}^{2}$. Given the presence of noise in the measurement, then, the dominant source of uncertainty in the measurement of $B_{0}$ by way of the vector component measurement technique described in Equation (2) is the uncertainty in the measurement of $B^{\prime }$:

(17) $$\begin{array}{c} B_{measured}^{\prime }=B^{\prime }\{1+\frac{2\delta B_{1}cos\left(\phi_{1}\right)}{B^{\prime }}\\ +2\sum_{k}\frac{\delta B_{k}\delta B_{k+2}}{B^{\prime 2}}\left[cos\left(\phi_{k}+\phi_{k+2}\right)+cos\left(\phi_{k}-\phi_{k+2}\right)\right]{\}}^{\frac{1}{2}} \end{array}$$

Equation (17) demonstrates that, in general, the uncertainty can reasonably be expected to decrease with increasing amplitude $B^{\prime }$ of the applied oscillating field in addition to benefiting from any filtering prior to the squaring operation that reduces the $\delta B_{k\neq 1}$ components. Additionally notable is that an increase in the feedback loop gain and improvement in phase response at kω will suppress any feedback loop contributions to the uncertainty shown in Equation (17). Though not explicit in Equation (17), each $\delta B_{k}$ includes uncertainty $\delta B_{k,noise}$ from the instrument noise as well as uncertainty $\delta B_{k,feedback}$ based on the finite gain of the closed-loop system and an offset $\delta B_{offset}$, which may arise from such effects as any noise rectification.

The error term $\delta B_{k,feedback}$ can be understood as follows. The closed-loop system includes a transfer function G(2πf) as a function of frequency f for the instrument and electronics along with a feedback transfer function K(2πf), resulting in a finite open-loop transfer function GK(2πf). The residual error $\delta B_{k,feedback}$ in the presence of applied oscillating field $B^{\prime }$ due to the finite response of the closed loop system is therefore proportional to the magnitude of the applied oscillating field:

(18) $$\begin{array}{c} \delta B_{k,feedback}=B^{\prime }\frac{1}{1+GK(k\omega )} \end{array}$$

In the limit that $\delta B_{k,feedback}\gg \delta B_{k,noise}$, the contribution to uncertainty in the measurement of $B^{\prime }$ arising from the feedback loop will dominate. As described in Equations (17) and (18), the uncertainty will no longer appreciably decrease with increasing amplitude $B^{\prime }$. Thus, for G(2πf)→fixed, an improvement in the feedback loop transfer function becomes the sole means of further reduction in uncertainty, which is the focus of this work. Note that for a 3-axis system, this condition will depend on the direction of the bias field relative to a respective applied oscillating field; the feedback uncertainty contribution will depend on the observed modulation of the scalar field imparted by the respective applied oscillating field. Comparing extreme cases in which the bias field is orthogonal to the applied oscillating field versus the case studied here, in which the applied oscillating field is along the bias field, Equation (2) demonstrates that in the extreme case, the applied oscillating field must be much larger than in the case studied here to meet the condition that $\delta B_{k,feedback}\gg \delta B_{k,noise}$.

## 3. Results

For each of the three feedback methods investigated in this work, 60 s of scalar magnetometer data were collected using each feedback loop method; in each case, an oscillating magnetic field was superimposed on the bias magnetic field by applying a modulating current through the same magnetic field coil that provides the bias field itself. These oscillating fields were applied at four frequencies (20 Hz, 200 Hz, 2 kHz, and 20 kHz) at each of four magnetic perturbation amplitudes (0.534, 5.34, 53.4, and 534 nT, corresponding to 3.74, 37.4, 374, and 3740 Hz perturbation amplitude in precession frequency units). The amplitudes of the applied oscillating fields were calibrated by way of measuring the change in spin precession frequency per unit drive signal input. For each data set, Equation (2) is used as the basis to solve for the vector component of the bias field that is oriented along the applied oscillating field. In this case, the bias field and oscillating field are co-aligned, simplifying the process of evaluating the accuracy of the vector field measurement as compared to the scalar magnetometer using this technique. In particular, if a result shows a high relative accuracy, the vector field component that is measured based on the ω and 2ω components of $M^{2}$ as shown in Equation (2) will be equal to the observed scalar field $B_{0}$.

**Figure 6:**
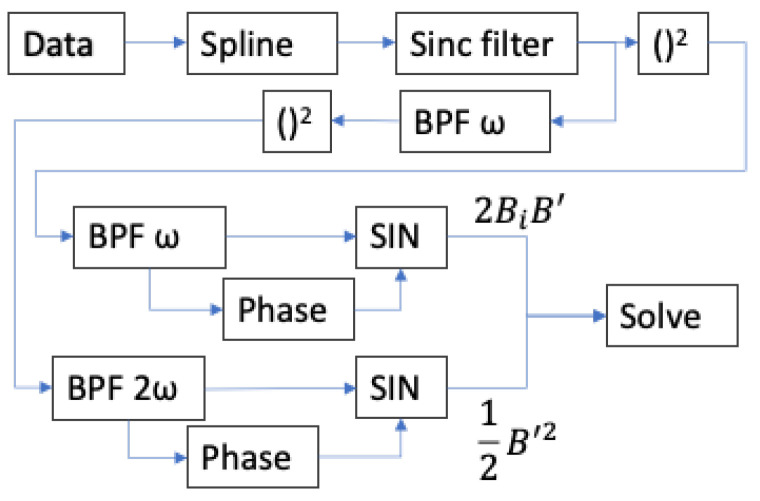
A top-level block diagram of the implemented vector calculation algorithm for a single axis, for each data set. The block labeled Spline represents cubic spline interpolation of the data for optimal sinc filtering (block labeled Sinc). After the sinc filter, the data are either immediately piecewise-squared (each data point in the time series is itself squared) or filtered first to suppress $\delta B_{k\neq 1}$ components (Equation (17)) and then piecewise squared. The blocks labeled BPF represent band-pass filters at ω and 2ω. The blocks labeled Phase represent phase detection to determine the appropriate demodulation phase (Equation (21)). The blocks labeled SIN represent sinusoidal demodulation, in which the signal is multiplied by a sine wave at the appropriate frequency and phase and then low-pass filtered to observe the low-frequency component of the output; these then feed into a solver block to measure the incident vector field ($B_{i}$) and the oscillating field ($B^{\prime }$) magnitude.

Each of the 48 data sets (four amplitudes at each frequency, four frequencies, and three methods) is analyzed by way of the algorithm shown in Figure 6 using MATLAB. The calculations shown in Figure 6 are implemented as follows. First, the scalar magnetometer data are upsampled using cubic spline interpolation (the “Spline” block in Figure 6) to increase the effective data rate of all data streams to a uniform pre-selected effective data rate, chosen such that every frequency of applied oscillating field is represented by an integer countdown of the effective data rate; this significantly simplifies the design of Sinc (in frequency) filters that may be applied to the data. The upsampled data are then filtered using a Sinc filter that is implemented as a simple moving average using the MATLAB command movmean(data, n), where n is the number of data points in the moving average. This filter suppresses undesired frequency components of *M*, which would lead to additional frequency mixing and corresponding uncertainty in the measurement of the 2ω component of $M^{2}$, such as noise in the vicinity of Nω, where *N* is an even integer up to a limit imposed by the sample rate. The magnitude part of the transfer function of the Sinc filter can be easily understood in a continuous-time approximation. The average over period *T* of cos(κt) and starting at an arbitrary time $t_{0}$ = 0 is simply a Sinc function:

(19) $$\begin{array}{c} \int_{0}^{T}\frac{cos(\kappa t)}{T}dt=\frac{sin(\kappa T)}{\kappa T}=Sinc\left(\kappa T\right) \end{array}$$

As noted in the discussion above regarding Equation (17), the suppression of $\delta B_{k\neq 1}$ components will minimize uncertainty in the measurement of $B^{\prime }$; thus, prior to squaring the magnetic field for the measurement of $B^{\prime }$, a bandpass filter at ω (i.e., k=1) is applied to suppress these undesired components in *M*. The $M^{2}$ data sets are further bandpass filtered at ω and 2ω as appropriate, then demodulated at the appropriate phase to measure values corresponding to the oscillating terms in Equation (2) so that a solution can be found for the measurement of the vector component of field along the oscillating field direction. In each case, the bandpass filter is designed using MATLAB’s built-in functionality for generating a minimum-order Chebyshev Type II filter; for maximum commonality of filter behavior across the range of frequencies, the filter bandwidth is kept at a constant fraction of the filter center frequency for both the pass-band and the stop-band, and the pass-band ripple and stop-band attenuation specifications remain constant across all filter instances.

The “Phase” block in Figure 6 refers to the calculation of the ideal phase for demodulation of $M^{2}$. Consider an applied magnetic field modulation component $2B_{0,i}B_{ac,i}sin\left(\omega_{i}t+\phi_{B}\right)$ (Equation (2)), where $\phi_{B}$ is an unknown phase relationship between the modulation signal as observed in the data and the start of the data set. The ideal demodulation signal to measure the amplitude $2B_{0,i}B_{ac,i}$ of the resulting oscillation will of course be to multiply the signal by $sin(\omega t+\phi_{B})$. The most precise possible value of $\phi_{B}$ can be calculated based on the entire data set—an advantage of post processing. Consider multiplication of the entire data set of a given $M^{2}$ separately by $cos(\omega_{i}t)$ and $sin(\omega_{i}t)$. Examining the effect on the $2B_{0,i}B_{ac,i}sin\left(\omega_{i}t+\phi_{B}\right)$ component of the signal and ignoring the amplitude for the moment in order to visually simplify the equations,

(20) $$\begin{array}{c} sin\left(\omega_{i}t\right)sin\left(\omega_{i}t+\phi_{B}\right)=\frac{1}{2}\left[cos\left(\phi \right)-cos\left(2\omega_{i}+\phi_{B}\right)\right]\\ cos\left(\omega_{i}t\right)sin\left(\omega_{i}t+\phi_{B}\right)=\frac{1}{2}\left[sin\left(\phi \right)-sin{\left(2\omega_{i}+\phi \right)}_{B}\right] \end{array}$$

Taking the mean of these outputs over the full data set (effectively eliminating the $2\omega_{i}$ components), one may solve for $\phi_{B}$:

(21) $$\begin{array}{c} \phi_{B}=tan^{-1}\frac{mean[cos\left(\omega_{i}t\right)*M^{2}\left(t\right)]}{mean[sin\left(\omega_{i}t\right)*M^{2}\left(t\right)]} \end{array}$$

This same technique for the extraction of the appropriate demodulation phase will apply to any signal of interest, and will allow the calculation of both the magnitude response and the (magnitude ∗ phase) response of an incident signal, the in-phase and quadrature components of the signal. In a real-time system, this calculation may be implemented by way of replacing the “mean” with appropriate low-pass filtering.

**Figure 7:**
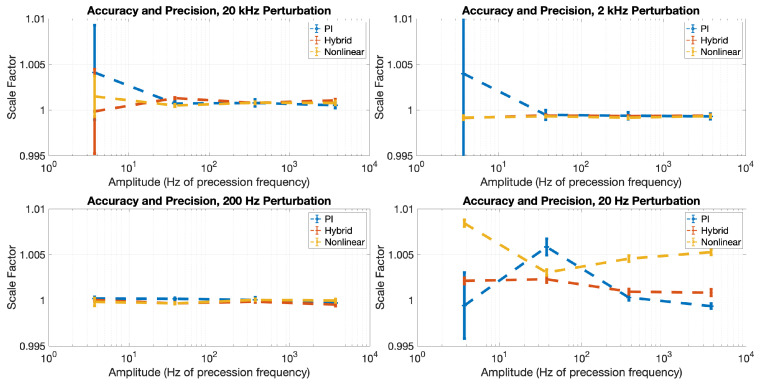
The relative accuracy of the oscillating field method compared to the scalar magnetometer (line plots with data points) and precision (error bars) of each method. The scale factor (Y axis) is the ratio between the measured vector field using the oscillating field method and the actual magnetic field as measured by the scalar magnetometer, while the precision is measured as the Allan deviation value of the measured vector time series at one second integration time.

Figure 7 shows the ratio (scale factor) between the vector component of magnetic field as measured using our vector measurement algorithm (Figure 6) and the magnetic field as measured by the scalar magnetometer, after correction for the gains of the filters shown in Figure 6. Ideally, the vector measurement algorithm will yield exactly the same result as the scalar measurement; in such a case, the scale factor would be exactly 1. As shown in Figure 7, the scale factor error for our measurement method is less than 1% in all cases (excluding error bars); in many instances, the scale factor is consistent with exactly 1 within three standard deviations.

**Figure 8:**
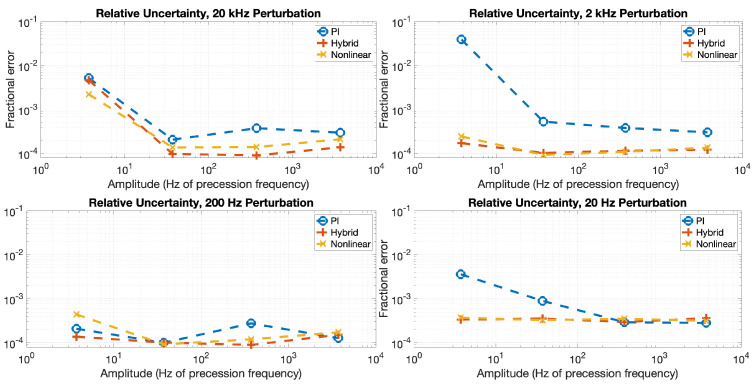
(color online) The fractional uncertainty of the measured static vector magnitudes, measured as the Allan deviation value at one second of integration time for each data set.

Recall from Equations (17) and (18) that it is specifically in the limit that $\delta B_{k,feedback}>\delta B_{k,noise}$ wherein an appreciable improvement in uncertainty based on the response of the feedback loop is expected. Figure 8 is consistent with this prediction; no clear advantage in precision is gained for the nonlinear or hybrid feedback methods over the PI method for any $B^{\prime }$ amplitude investigated herein at 20 Hz and 200 Hz, where |GK|≫1 for all three methods. Meanwhile, at 2 kHz and 20 kHz, the precision follows the expected progression of $\delta B_{k,PI}>\delta B_{k,Nonlinear}>\delta B_{k,Hybrid}$ based on the respective closed-loop response characteristics, with the difference between the nonlinear and hybrid methods being most clear at the highest frequency. It is, therefore, concluded that in this work, magnetometer noise dominates the precision of the vector measurement method shown in Equation (2) when $\delta B_{k,feedback}<\delta B_{k,noise}$, magnetic measurement noise dominates the scale factor error, and the chosen feedback method will dominate the relative uncertainty at larger $B^{\prime }$ and larger ω, where $\delta B_{k,feedback}>\delta B_{k,noise}$.

## 4. Conclusions

This work examined and analyzed the comparative suitability of three different feedback methods for the closed-loop operation of a Bell–Bloom magnetometer [33,34] operating in Earth-field-scale magnetic fields and driven by intensity-modulated optical pump light [20] with the intent of measuring the vector components of the incident magnetic field by way of applying oscillating magnetic fields [35]. The present work takes a first step toward 3-axis vector measurement by applying a single oscillating field along the bias field so that the effects of the feedback method on the relative accuracy and precision of the vector component measurement can be robustly evaluated in a simple and straightforward manner. The investigated feedback methods include proportional integral (PI), nonlinear, and hybrid self-oscillator feedback methods.

This work demonstrated, in accordance with Equations (17) and (18), that with a combination of sufficiently large amplitude and sufficiently high frequency applied oscillating magnetic field, appreciable improvements in measurement uncertainty can only be realized by way of improvements in the feedback loop response. In this work, these improvements are demonstrated when using feedback methods which capture a greater portion of the nonlinear response of the instrument to the increased perturbation amplitude and frequency (Equation (5)). It is further important to note that the method outlined herein for the measurement of the vector magnetic field components [35] draws its accuracy from the accuracy of the scalar magnetometer itself in addition to any accuracy considerations in the feedback method and the vector calculation process (Figure 6). Thus, to meet any particular absolute accuracy specification for the measurement of the vector components of the incident magnetic field, the scalar magnetometer must be at least as accurate as the desired vector accuracy.

In future work, the vector measurement and feedback methods described herein may be extended to 3-axis vector magnetic field measurement. Further, the vector component measurement algorithms may be implemented in real time for the active measurement of the vector components of the magnetic field. Additionally, as described above, the error signal δϕ that drives the feedback loop can be updated to improve the response in a wide variety of magnetic field magnitudes. Finally, the magnetometer performance may be evaluated in an unshielded magnetic environment.

## Data Availability

Data and MATLAB code available at https://uwmadison.box.com/s/j1rdssru93jjyum7tj3yaoas15kugx0h (accessed 28 March 2023). To process data, download all data and MATLAB code files (.m files) to a single folder and run the code Data_to_Vector_Time_series.m from the same folder.

## References

[1] Strasburger J., Cheulkar B., Wakai R. (2008). Magnetocardiography for fetal arrhythmias. Heart Rhythm..

[2] Hämäläinen M., Hari R., Ilmoniemi R., Knuutila J., Lounasmaa O. (1993). Magne-toencephalography—Theory, instrumentation, and applications to noninvasive studies of the working hu-man brain. Rev. Mod. Phys..

[3] Sulai I.A., DeLand Z.J., Bulatowicz M.D., Wahl C.P., Wakai R.T., Walker T.G. (2019). Characterizing atomic magnetic gradiometers for fetal magnetocardiography. Rev. Sci. Instrum..

[4] Boto E., Shah V., Hill R.M., Rhodes N., Osborne J., Doyle C., Holmes N., Rea M., Leggett J., Bowtell R. (2022). Triaxial detection of the neuromagnetic field using optically-pumped mag-netometry: Feasibility and application in children. Neuroimage.

[5] Rea M., Boto E., Holmes N., Hill R., Osborne J., Rhodes N., Leggett J., Rier L., Bowtell R., Shah V. (2022). A 90-channel triaxial magnetoencephalography system using optically pumped magnetometers. Ann. N. Y. Acad. Sci..

[6] Klotz T., Gizzi L., Röhrle O. (2022). Investigating the spatial resolution of EMG and MMG based on a systemic multi-scale model. Biomech. Model. Mechanobiol..

[7] Hoshino Y., Kawabata S., Adachi Y., Watanabe T., Sekihara K., Sasaki T., Hashimoto J., Fujita K., Nimura A., Okawa A. (2022). Magnetoneurography as a novel functional imaging technique for the ulnar nerve at the elbow. Clin. Neurophysiol..

[8] Harry G., Jin I., Paik H., Stevenson T., Wellstood F. (2000). Two-stage superconduct-ing-quantum-interference-device amplifier in a high-Q gravitational wave transducer. Appl. Phys. Lett..

[9] Lorenz R., Jones J., Wu J. Mars magnetometry from a tumbleweed rover. Proceedings of the 2003 IEEE Aerospace Conference Proceedings (Cat. No.03TH8652).

[10] Afach S., Buchler B., Budker D., Dailey C., Derevianko A., Dumont V., Figueroa N., Gerhardt I., Grujić Z., Guo H. (2021). Search for topological defect dark matter with a global network of optical magnetometers. Nat. Phys..

[11] Horbury T., O’Brien H., Carrasco Blazquez I., Bendyk M., Brown P., Hudson R., Evans V., Oddy T., Carr C., Beek T. (2020). The Solar Orbiter magnetometer. Astron. Astrophys..

[12] Vo C.K., Staples S.G.H., Cowell D.M., Varcoe B.T.H., Freear S. (2020). Determining the Depth and Loca-tion of Buried Pipeline by Magnetometer Survey. J. Pipeline Syst. Eng. Pract..

[13] Koss P., Durmaz A., Blug A., Laskin G., Pawar O., Thiemann K., Bertz A., Straub T., Elsässer C. (2022). Optically Pumped Magnetometer Measuring Fatigue-Induced Damage in Steel. Appl. Sci..

[14] Canciani A., Raquet J. (2016). Absolute Positioning Using the Earth’s Magnetic Anomaly Field. NAVIGATION.

[15] Dang H., Maloof A., Romalis M. (2010). Ultrahigh sensitivity magnetic field and magnetization measurements with an atomic magnetometer. Appl. Phys. Lett..

[16] Wang K., Zhang K., Zhou B., Lu F., Zhang S., Yan Y., Wang W., Lu J. (2022). Triaxial closed-loop measurement based on a single-beam zero-field optically pumped magnetometer. Front. Phys..

[17] Yan Y., Lu J., Zhang S., Lu F., Yin K., Wang K., Zhou B., Liu G. (2022). Three-axis closed-loop optically pumped magnetometer operated in the SERF regime. Opt. Express.

[18] Lu F., Lu J., Li B., Yan Y., Zhang S., Yin K., Ye M., Han B. (2022). Triaxial Vector Operation in Near-Zero Field of Atomic Magnetometer With Femtotesla Sensitivity. IEEE Trans. Instrum. Meas..

[19] Huang H.C., Dong H.F., Hu X.Y., Chen L., Gao Y. (2015). Three-axis atomic magnetom-eter based on spin precession modulation. Appl. Phys. Lett..

[20] Gerginov V., Krzyzewski S., Knappe S. (2017). Pulsed operation of a miniature scalar optically pumped magnetometer. J. Opt. Soc. Am. B.

[21] Seltzer S., Romalis M. (2004). Unshielded three-axis vector operation of a spin-exchange-relaxation-free atomic magnetometer. Appl. Phys. Lett..

[22] Perry A., Bulatowicz M., Larsen M., Walker T., Wyllie R. (2020). All-optical intrinsic atomic gradiometer with sub-20 fT/cm/rtHz sensitivity in a 22 *μ*T earth-scale magnetic field. Opt. Express.

[23] Oelsner G., IJsselsteijn R., Scholtes T., Krüger A., Schultze V., Seyffert G., Werner G., Jäger M., Chwala A., Stolz R. (2022). Integrated Optically Pumped Magnetometer for Measurements within Earth’s Magnetic Field. Phys. Rev. Appl..

[24] Schönau T., Schmelz M., Zakosarenko V., Stolz R., Meyer M., Anders S., Fritzsch L., Meyer H. (2013). SQUID-based setup for the absolute measurement of the Earth’s magnetic field. Supercond. Sci. Technol..

[25] Budker D., Romalis M. (2007). Optical Magnetometry. Nat. Phys..

[26] Grosz A., Haji-Sheikh M., Mukhopadhyay S. (2016). High Sensitivity Magnetometers. Smart Sensors, Measurement and Instrumentation.

[27] Thiele T., Lin Y., Brown M.O., Regal C.A. (2018). Self-Calibrating Vector Atomic Magnetometry through Microwave Polarization Reconstruction. Phys. Rev. Lett..

[28] Soheilian A., Ranjbaran M., Tehranchi M.M. (2020). Position and Direction Tracking of a Magnetic Object Based on an Mx-Atomic Magnetometer. Sci. Rep..

[29] National Centers for Environmental Information, National Oceanic and Atmospheric Administration (NOAA), Enhanced Magnetic Model (EMM). https://www.ngdc.noaa.gov/geomag/EMM/.

[30] Lee W., Lucivero V., Romalis M., Limes M., Foley E., Kornack T. (2021). Heading errors in all-optical al-kali-metal-vapor magnetometers in geomagnetic fields. Phys. Rev. A.

[31] Sui Y., Li G., Wang S., Lin J. (2014). Compact fluxgate magnetic full-tensor gradiometer with spher-ical feedback coil. Rev. Sci. Instrum..

[32] Huang Y., Wu L., Li D. (2015). Theoretical Research on Full Attitude Determination Using Geomagnetic Gradient Tensor. J. Navig..

[33] Bell W.E., Bloom A.L. (1957). Optical detection of magnetic resonance in alkali metal vapor. Phys. Rev..

[34] Bell W.E., Bloom A.L. (1961). Optically driven spin precession. Phys. Rev. Lett..

[35] Walker T.G., Bulatowicz M.D. (2022). Synchronous Light-Pulse Atomic Magnetometer System. U.S. Patent.

[36] Gravrand O., Khokhlov A., Le Mouël J.L., Léger J.M. (2001). On the calibration of a vectorial 4He pumped magnetometer. Earth Planets Space.

[37] Bulatowicz M. (2012). Temperature System with Magnetic field Suppression. U.S. Patent.

[38] Kitching J. (2018). Chip-scale atomic devices. Appl. Phys. Rev..

